# A circular RNA from *APC* inhibits the proliferation of diffuse large B-cell lymphoma by inactivating Wnt/β-catenin signaling via interacting with TET1 and miR-888

**DOI:** 10.18632/aging.102122

**Published:** 2019-10-13

**Authors:** Yanping Hu, Yixun Zhao, Chao Shi, Pengfei Ren, Bing Wei, Yongjun Guo, Jie Ma

**Affiliations:** 1 Department of Molecular Pathology, The Affiliated Cancer Hospital of Zhengzhou University, Henan Cancer Hospital, Zhengzhou, Henan 450008, P.R. China; 2 Endoscopic Center, The Affiliated Cancer Hospital of Zhengzhou University, Henan Cancer Hospital, Zhengzhou, Henan 450008, P.R. China

**Keywords:** circular RNA, diffuse large B-cell lymphoma, circ-APC, biomarker

## Abstract

Circular RNA (circRNA), a type of non-coding RNA, can promote or suppress tumorigenesis. To investigate the involvement of circRNA in diffuse large B-cell lymphoma (DLBCL), we performed a circRNA microarray analysis on paired DLBCL and normal tissues. We identified a novel and highly stable circRNA originating from the back-splicing of *APC* exon 7 to exon 14, circ-APC (hsa_circ_0127621), which was downregulated in DLBCL tissues, cell lines and plasma. In gain-of-function experiments, ectopic expression of circ-APC inhibited DLBCL cell proliferation *in vitro* and tumor growth *in vivo*. Cytoplasmic circ-APC functioned as a sponge for miR-888, thus post-transcriptionally upregulating *APC* by alleviating the repressive effects of miR-888 on this gene. Further, nuclear circ-APC bound to the *APC* promoter and recruited the DNA demethylase TET1, thereby transcriptionally upregulating *APC*. Upon its upregulation, APC dampened the canonical Wnt/β-catenin signaling pathway by reducing the accumulation of β-catenin in the nucleus, thereby retarding DLBCL growth. Clinically, circ-APC was found to be an effective diagnostic and prognostic biomarker for patients with DLBCL. Our study suggests that circ-APC is a novel proliferation inhibitor, and that restoring circ-APC expression may be a promising therapeutic approach for DLBCL patients.

## INTRODUCTION

Diffuse large B-cell lymphoma (DLBCL), a highly aggressive and heterogeneous type of malignant tumor, is the most common form of non-Hodgkin lymphoma, accounting for almost one-third of all cases [1]. Currently, the standard first-line treatment for DLBCL is rituximab combined with chemotherapy (cyclophosphamide/ doxorubicin/vincristine/prednisone), which is referred to as the R-CHOP regimen [2]. The prognosis of DLBCL patients remains unsatisfactory, especially among those with R-CHOP insensitivity and disease recurrence, and the five-year survival rate is less than 30% [3]. Therefore, there is an urgent need to clarify the underlying pathogenesis of DLBCL, as this may provide new targets for clinical diagnosis and treatment.

Circular RNA (circRNA) is a novel type of non-coding RNA having no 5′ cap or 3′ poly-A tail, but having a covalently closed loop structure generated by head-to-tail splicing. Due to this structure, circRNA is resistant to exonucleases and is more stable than linear non-coding RNAs such as microRNA (miRNA) and long non-coding RNA [4]. CircRNA production has long been considered a low-probability event during transcription, so circRNAs have been regarded as “junk sequences” without definitive biological functions [5]. However, this notion has completely changed with the advent of high-throughput sequencing. Numerous studies have confirmed that circRNA is abundant, highly conserved and capable of gene regulation in eukaryotes [6]. Moreover, circRNA is known to be involved in the occurrence, development and progression of human diseases, especially cancer [7]. A circRNA may function as an oncogene or a tumor suppressor, thus altering tumor proliferation, migration, invasion, angiogenesis and so on. However, it is still unknown whether circRNA participates in the malignancy of DLBCL.

The mechanism by which a circRNA functions mainly depends on its subcellular localization. Cytoplasmic circRNA can function as an “miRNA sponge”, bind to proteins and even translate proteins. Extensive studies have confirmed that circRNAs can efficiently sponge miRNAs to reduce their inhibitory effects on their target genes, in what is known as the competitive endogenous RNA mechanism. For example, ciRS-7 was reported to contain more than 70 selectively conserved binding sites for miR-7 and to elevate the expression of miR-7 targets [8, 9]. On the other hand, nuclear circRNA can function as a cis-acting transcriptional regulator by binding to proteins. For instance, circ-β-catenin was reported to activate β-catenin by binding to DDX3, thereby facilitating tumorigenesis and tumor aggressiveness [10].

In the current study, we identified a novel DLBCL-related circRNA (circ-APC) through a circRNA microarray. We then explored the expression, biological function, underlying mechanism and clinical significance of circ-APC in DLBCL.

## RESULTS

### Characterization of circ-APC in DLBCL

**Figure 1:**
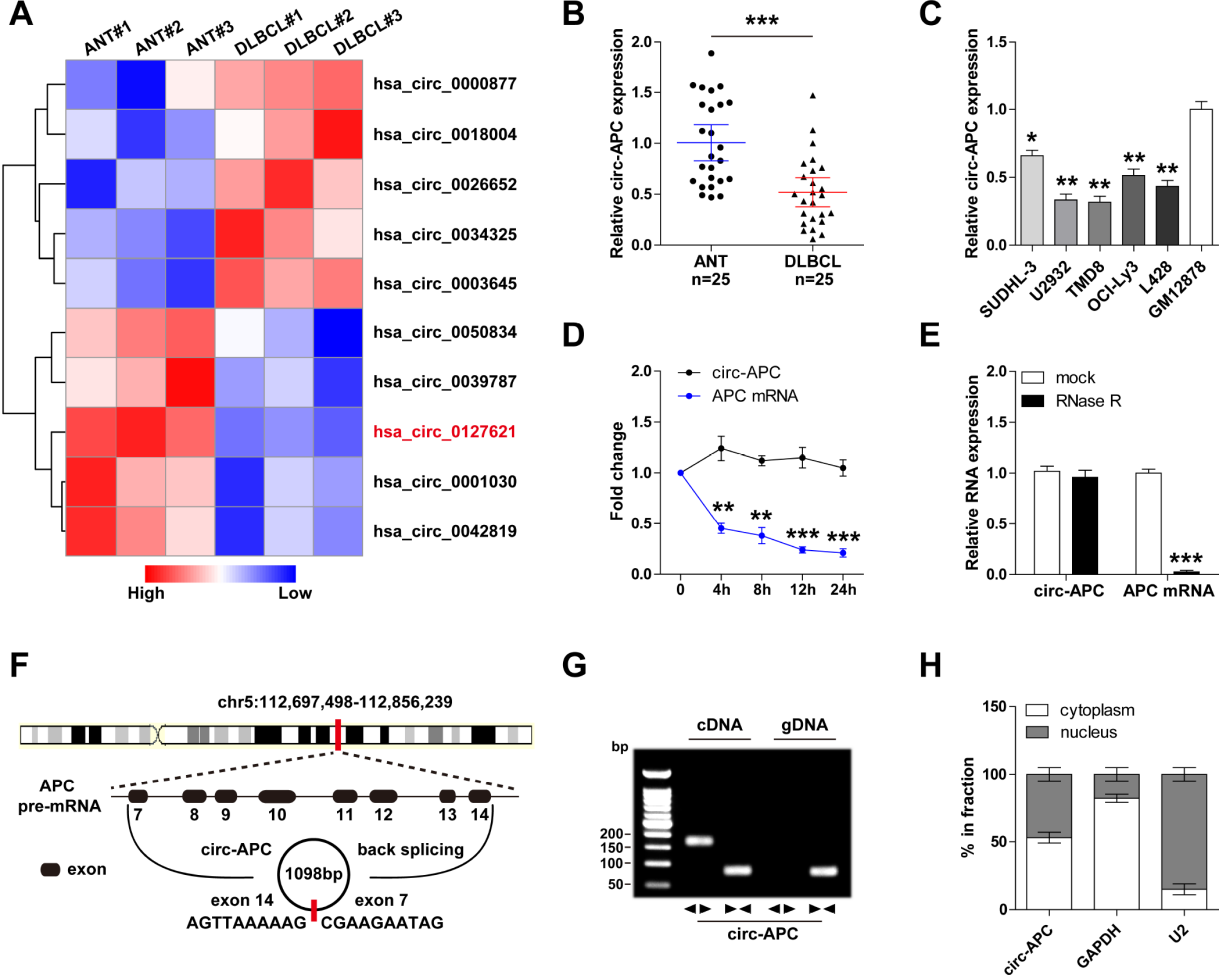
**Identification of circ-APC as a DLBCL-related circRNA.** (**A**) Heat map of the top five differentially expressed circRNAs in three paired DLBCL and normal tissues. (**B** and **C**) qRT-PCR analysis of circ-APC expression in DLBCL tissues and cells. (**D**) qRT-PCR analysis of circ-APC and *APC* mRNA expression after treatment with actinomycin D for the indicated times. (**E**) qRT-PCR analysis of circ-APC and *APC* mRNA expression after treatment with RNase R. (**F**) Schematic diagram of the origin of circ-APC. (**G**) RT-PCR analysis of circ-APC expression with convergent and divergent primers for cDNA or gDNA. (**H**) qRT-PCR analysis of cytoplasmic and nuclear circ-APC expression. *p < 0.05, **p < 0.01, ***p < 0.001.

To search for differentially expressed circRNAs in DLBCL, we analyzed the circRNA microarray expression profiles of three pairs of DLBCL and para-carcinoma tissues (Figure 1A). We selected circ-APC (hsa_circ_ 0127621) for further investigation because its expression differed the most between DLBCL and para-carcinoma tissues. A quantitative real-time PCR (qRT-PCR) analysis demonstrated that circ-APC was significantly downregulated in DLBCL tissues compared with adjacent normal tissues, consistent with the findings of the circRNA microarray (Figure 1B). Likewise, circ-APC expression was lower in five DLBCL cell lines than in GM12878 normal human B lymphocytes (Figure 1C).

We then tested the stability of circ-APC by using actinomycin D, a transcription inhibitor. As shown in Figure 1D, the half-life of *APC* mRNA was less than 4 hours, while that of circ-APC was greater than 24 hours, suggesting that circ-APC is highly stable. Moreover, the exoribonuclease RNase R effectively degraded almost all the linear *APC* mRNA, but had no effect on circ-APC, implying that circ-APC harbors a loop structure (Figure 1E).

Through sequence alignment, we found that circ-APC was generated through the back-splicing of linear *APC* exon 7 to exon 14, and that the mature spliced full-length sequence was 1098 bp long (Figure 1F). This was confirmed by reverse transcriptase (RT)-PCR with divergent primers for the junction sequence of circ-APC (Figure 1G).

A qRT-PCR analysis of cytoplasmic and nuclear cellular fractions revealed that circ-APC was relatively evenly distributed between the cytoplasm and nucleus (Figure 1H). These findings suggested that circ-APC is a highly stable circRNA that may be involved in DLBCL tumorigenesis.

### Exogenous expression of circ-APC inhibits DLBCL cell proliferation both *in vitro* and *in vivo*

**Figure 2:**
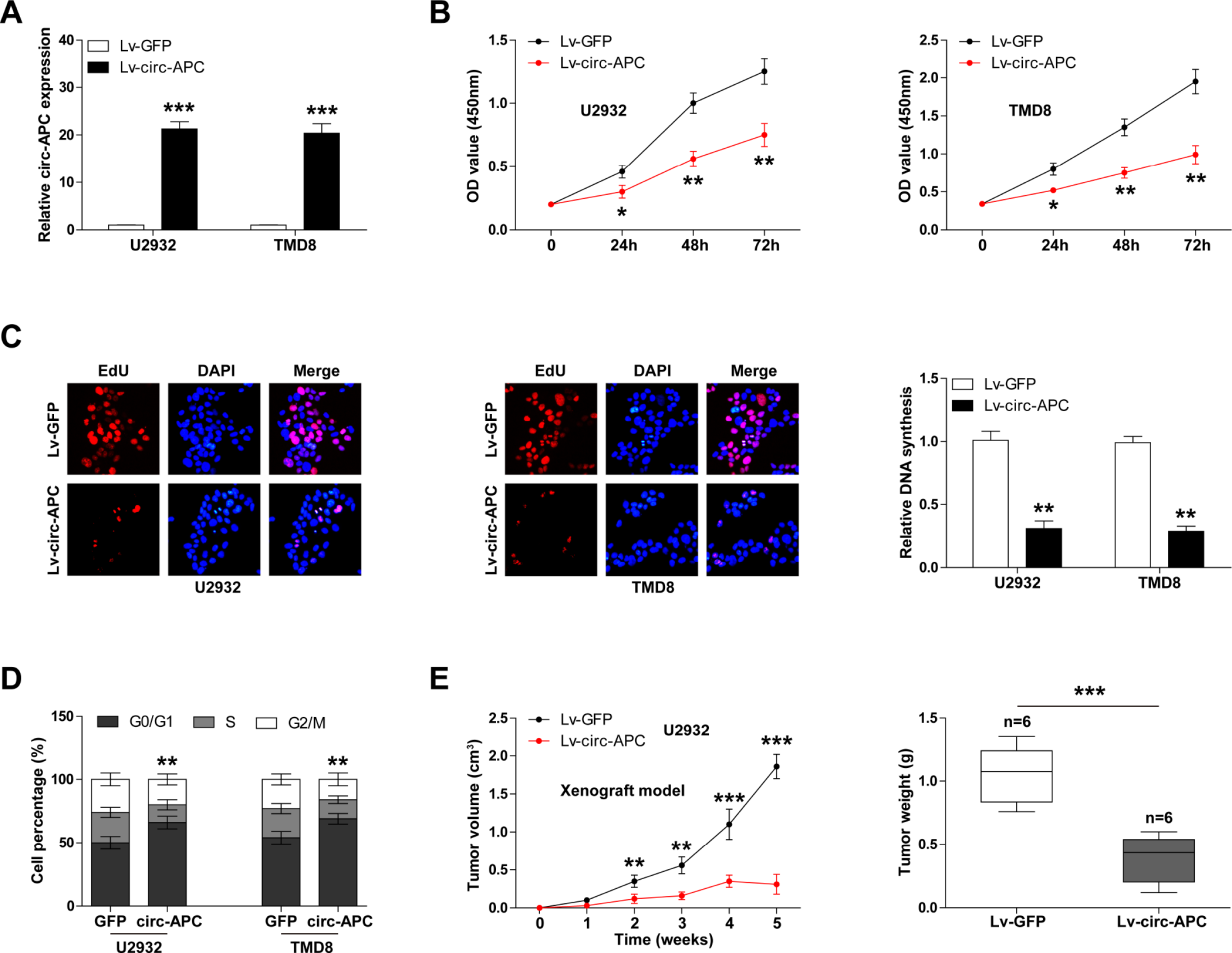
**Exogenous expression of circ-APC inhibits DLBCL cell proliferation both *in vitro* and *in vivo*.** (**A**) qRT-PCR analysis of circ-APC expression in U2932 and TMD8 cells infected with a circ-APC-overexpressing lentiviral vector. (**B**) CCK-8 assay for DLBCL cell viability at the indicated times. (**C**) EdU assay to determine the DNA synthesis rate in control or stably circ-APC-overexpressing U2932 and TMD8 cells. Nuclei were stained by DAPI. (**D**) Cell cycle analysis (G0/G1, S, G2/M phase) in control or stably circ-APC-overexpressing U2932 and TMD8 cells. (**E**) The volumes and weights of the subcutaneous tumors in the control and circ-APC-overexpressing groups (n=6 per group). *p < 0.05, **p < 0.01, ***p < 0.001.

To explore the biological function of circ-APC, we used a lentiviral vector to construct stably circ-APC-overexpressing DLBCL cell lines. A qRT-PCR analysis demonstrated that circ-APC expression was upregulated approximately 20-fold in the circ-APC-overexpressing U2932 and TMD8 cell lines (Figure 2A).

We then evaluated the effects of circ-APC on the proliferation of DLBCL cells. A Cell Counting Kit-8 (CCK-8) assay revealed that cell viability was dramatically lower in circ-APC-overexpressing U2932 and TMD8 cells than in control cells (Figure 2B). Likewise, an EdU assay indicated that ectopic expression of circ-APC significantly impaired DNA synthesis (Figure 2C). Moreover, circ-APC overexpression increased the number of cells that were arrested at the G0/G1 phase (Figure 2D).

To test whether circ-APC could also reduce DLBCL cell proliferation *in vivo*, we established a xenograft model by subcutaneously inoculating nude mice with U2932 cells (n=6 per group). The volumes and weights of the subcutaneous tumors in the circ-APC-overexpressing group were significantly smaller than those in the control group (Figure 2E). These *in vitro* and *in vivo* experiments indicated that circ-APC is a proliferation inhibitor in DLBCL.

### Circ-APC elevates the expression of its host gene *APC* both *in vitro* and *in vivo*

**Figure 3:**
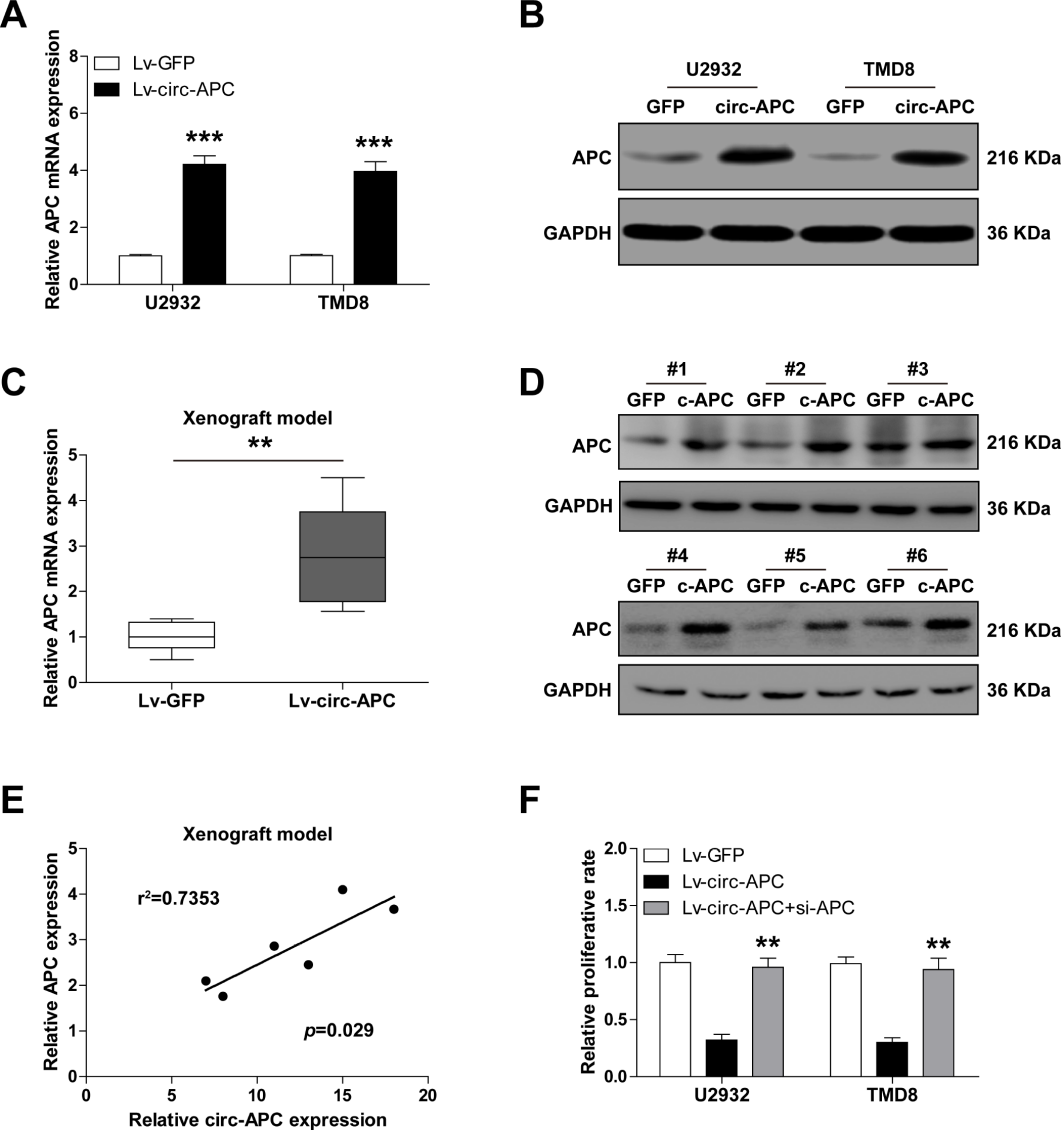
**Circ-APC significantly increases *APC* expression in DLBCL.** (**A** and **B**) qRT-PCR and Western blot analyses of *APC* expression in control or stably circ-APC-overexpressing U2932 and TMD8 cells. (**C** and **D**) qRT-PCR and Western blot analyses of *APC* expression in the subcutaneous tumors of nude mice injected with control or stably circ-APC-overexpressing U2932 cells. (**E**) The correlation between circ-APC and *APC* expression in the above subcutaneous tumors. (**F**) Cell proliferation rate in stably circ-APC-overexpressing U2932 and TMD8 cells transfected with si-APC. **p < 0.01, ***p < 0.001.

Given that circRNAs can alter the expression of their host genes and thereby participate in disease progression [11], we wondered whether circ-APC upregulated *APC*. As expected, overexpression of circ-APC remarkably increased *APC* mRNA and protein expression in both U2932 and TMD8 cells (Figure 3A, 3B). Importantly, the same effects on *APC* expression were observed in the tumors of the xenografted mice injected with circ-APC-overexpressing U2932 cells (Figure 3C, 3D). There was a strong positive correlation between circ-APC expression and *APC* expression in the circ-APC-overexpressing subcutaneous tumors (r^2^=0.7353, *p*=0.029, n=6) (Figure 3E).

To determine whether the inhibitory effects of circ-APC on cell proliferation depended on its ability to upregulate *APC,* we knocked down *APC* in U2932 and TMD8 cells. Silencing of *APC* almost completely reversed the suppressive effects of circ-APC on cell proliferation (Figure 3F). These results suggested that circ-APC positively regulates *APC* expression in DLBCL.

### Circ-APC is an efficient sponge for miR-888 in DLBCL

**Figure 4:**
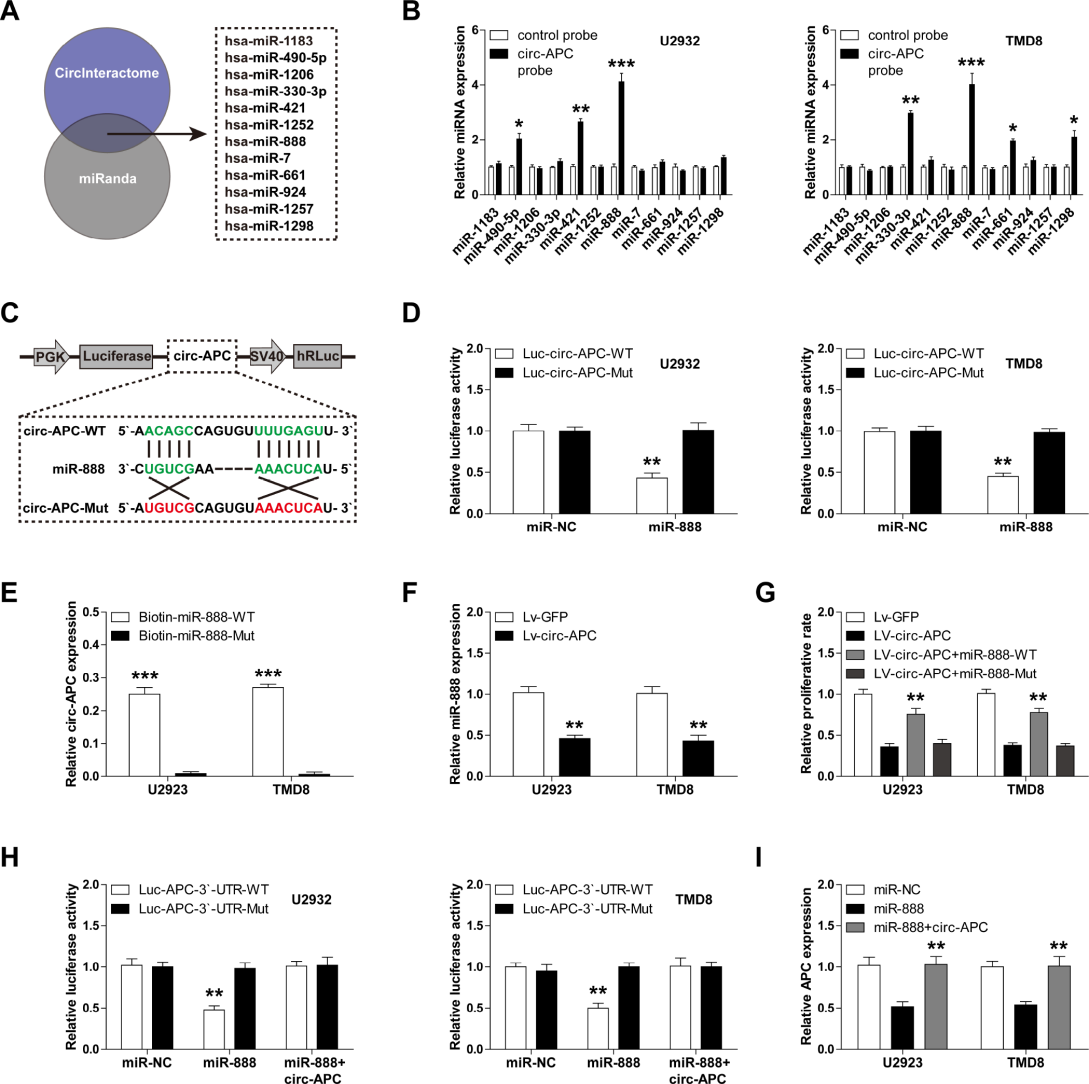
**Circ-APC can sponge miR-888 in DLBCL.** (**A**) The CircInteractome and miRanda online tools identified 12 miRNAs with putative binding sites for both circ-APC and *APC*. (**B**) RNA pull-down assay with a control or circ-APC probe in U2932 and TMD8 cells, followed by qRT-PCR analysis of the 12 miRNAs identified above. (**C**) Schematic diagram of the circ-APC luciferase reporter vector with a wild-type or mutant miR-888 binding site. (**D**) Luciferase reporter assay in U2932 and TMD8 cells co-transfected with the wild-type or mutant circ-APC luciferase vector and control miRNA or miR-888 mimics. (**E**) RNA pull-down assay in U2932 and TMD8 cells transfected with wild-type or mutant miR-888 mimics, followed by qRT-PCR analysis of circ-APC expression. (**F**) qRT-PCR analysis of miR-888 expression after circ-APC overexpression. (**G**) Cell proliferation rate in stably circ-APC-overexpressing U2932 and TMD8 cells transfected with wild-type or mutant miR-888 mimics. (**H**) Luciferase reporter assay in control or circ-APC-overexpressing U2932 and TMD8 cells co-transfected with the wild-type or mutant *APC* 3′-UTR luciferase vector and control miRNA or miR-888 mimics. (**I**) qRT-PCR analysis of *APC* expression in control or circ-APC-overexpressing U2932 and TMD8 cells transfected with control miRNA or miR-888 mimics. *p < 0.05, **p < 0.01, ***p < 0.001.

Cytoplasmic circRNA can function as a competitive endogenous RNA that governs gene expression by sponging miRNA [12]. Considering that about 50% of circ-APC was present in the cytoplasm, we speculated that circ-APC might regulate *APC* expression by this mechanism. Using the CircInteractome and miRanda online tools, we found a total of 12 miRNAs (miR-1183, miR-490-5p, miR-1206, miR-330-3p, miR-421, miR-1252, miR-889, miR-7, miR-661, miR-924, miR-1257 and miR-1298) with putative binding sites for both circ-APC and *APC* (Figure 4A).

To determine which miRNA is involved in the circ-APC/*APC* axis, we performed an RNA pull-down assay with a biotin-labeled circ-APC probe. Only miR-888 was abundantly pulled down by circ-APC in both U2932 and TMD8 cells (Figure 4B). We then conducted a luciferase reporter assay by inserting a wild-type or mutant full-length circ-APC sequence downstream of luciferase (Figure 4C). As shown in Figure 4D, overexpression of miR-888 significantly reduced the luciferase activity of the wild-type reporter, but did not affect the activity of the mutant reporter. To confirm the binding between circ-APC and miR-888, we performed an RNA pull-down assay in U2932 and TMD8 cells transfected with biotin-labeled wild-type or mutant miR-888 mimics. Circ-APC was effectively enriched by wild-type miR-888, but not by mutant miR-888 (Figure 4E).

To assess the involvement of miR-888 in DLBCL, we examined its expression in DLBCL cell lines, and found that it was remarkably upregulated (Supplementary Figure 1). However, circ-APC overexpression significantly reduced miR-888 expression (Figure 4F). To determine whether the anti-proliferative effects of circ-APC in DLBCL cells depended on its ability to downregulate miR-888, we transfected circ-APC-overexpressing U2932 and TMD8 cells with wild-type or mutant miR-888 mimics. Ectopic expression of wild-type miR-888, but not mutant miR-888, partially reversed the suppressive effects of circ-APC overexpression on DLBCL cell proliferation (Figure 4G).

Next, we tested whether *APC* was a target of miR- 888 by performing a luciferase reporter assay. While miR-888 overexpression did not alter the luciferase activity of a mutant *APC* 3′-UTR reporter, it dramatically reduced the activity of a wild-type reporter (Figure 4H). Circ-APC overexpression completely blocked this repression in both U2932 and TMD8 cells (Figure 4H). Consistently, qRT-PCR analysis revealed that miR-888 overexpression reduced *APC* expression, whereas exogenous circ-APC expression effectively restored *APC* expression (Figure 4I). These findings demonstrated that circ-APC increases *APC* expression by sponging and inhibiting miR-888 in DLBCL.

### Circ-APC physically binds to the *APC* promoter and recruits DNA demethylase TET1 in DLBCL

**Figure 5:**
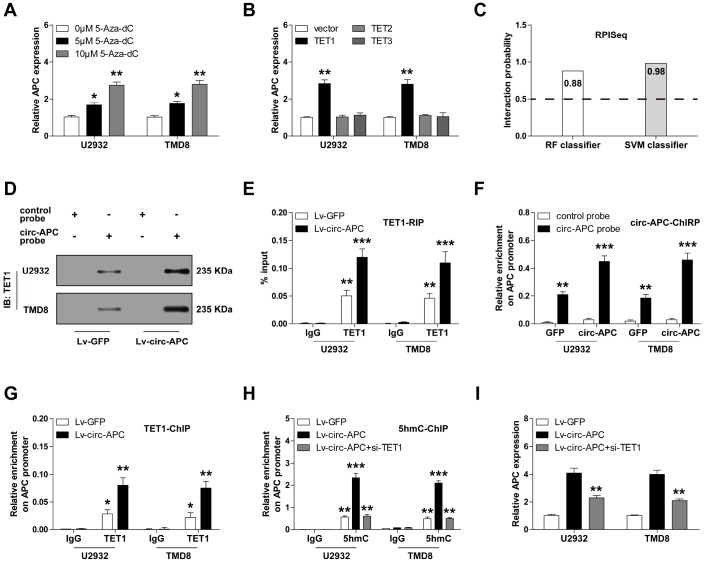
**Circ-APC recruits TET1 to the *APC* promoter to enhance the transcription of *APC* in DLBCL.** (**A**) qRT-PCR analysis of *APC* expression in U2932 and TMD8 cells treated with the indicated concentrations of 5-Aza-dC. (**B**) qRT-PCR analysis of *APC* expression in U2932 and TMD8 cells transfected with a *TET1*, *TET2* or *TET3* overexpression vector. (**C**) RPISeq online tool predicting the probability of binding between circ-APC and TET1. (**D**) RNA pull-down assay with a control or circ-APC probe, followed by Western blot analysis of TET1 expression. (**E**) RNA immunoprecipitation assay with an IgG or TET1 antibody, followed by qRT-PCR analysis of circ-APC expression. (**F**) ChIRP assay with a control or circ-APC probe, followed by qRT-PCR analysis of the *APC* promoter region. (**G** and **H**) ChIP assay with an IgG, TET1 or 5hmC antibody, followed by qRT-PCR analysis of the *APC* promoter region in U2932 and TMD8 cells after the indicated transfection. (**I**) qRT-PCR analysis of *APC* expression in stably circ-APC-overexpressing U2932 and TMD8 cells transfected with si-TET1. *p < 0.05, **p < 0.01, ***p < 0.001.

APC is a well-known tumor suppressor that is frequently inactivated due to hypermethylation of its promoter region [13]. We confirmed this notion by treating DLBCL cells with 5-Aza-dC, an inhibitor of DNA methyltransferases. As shown in Figure 5A, 5-Aza-dC dose-dependently increased *APC* expression in both U2932 and TMD8 cells.

Given that DNA hypermethylation can be attributed to reduced demethylase activity, we then investigated whether reduced demethylase expression could be responsible for the repression of *APC*. The ten-eleven translocation (TET) family of demethylases includes three members: TET1, TET2 and TET3 [14]. *TET1* expression was significantly lower in U2932 and TMD8 cells than in normal GM12878 cells (Supplementary Figure 2). Accordingly, overexpression of TET1, but not TET2 or TET3, significantly upregulated *APC* mRNA expression (Figure 5B).

Recent studies have suggested that circRNA can regulate gene expression by binding to proteins [15]. Thus, we wondered whether circ-APC could directly bind to TET1. The RPISeq online tool predicted a high interaction possibility between circ-APC and TET1 (RF classifier = 0.88, SVM classifier = 0.98, predictions with probabilities > 0.5 were considered “positive”) (Figure 5C). To confirm this prediction, we performed an RNA pull-down assay. TET1 was enriched by a circ-APC probe compared with a control probe, and this phenomenon was more pronounced in circ-APC-overexpressing U2932 and TMD8 cells (Figure 5D). Similarly, in an RNA immunoprecipitation assay, a TET1 antibody, but not an IgG antibody, abundantly precipitated circ-APC (Figure 5E).

In light of the binding between circ-APC and TET1, we wondered whether circ-APC could recruit TET1 to the *APC* promoter. First, we performed a ChIRP assay in U2932 and TMD8 cells, which demonstrated that circ-APC could bind directly to the *APC* promoter (Figure 5F). We then evaluated the occupation of the *APC* promoter by TET1, and found a greater amount of TET1 after exogenous circ-APC expression (Figure 5G). As TET1 oxidizes 5mC to 5hmC, we also assessed 5hmC levels on the *APC* promoter. Consistently, the 5hmC levels on the *APC* promoter were higher in circ-APC-overexpressing U2932 and TMD8 cells than in control cells, whereas this effect was completely abolished by *TET1* silencing (Figure 5H). In addition, knockdown of *TET1* partly counteracted the increase in *APC* expression following circ-APC overexpression (Figure 5I). These results demonstrated that DNA demethylation by TET1 contributes to the upregulation of *APC* by circ-APC in DLBCL.

### Circ-APC inactivates canonical Wnt/β-catenin signaling through the miR-888/*APC* and TET1/*APC* axes in DLBCL

**Figure 6:**
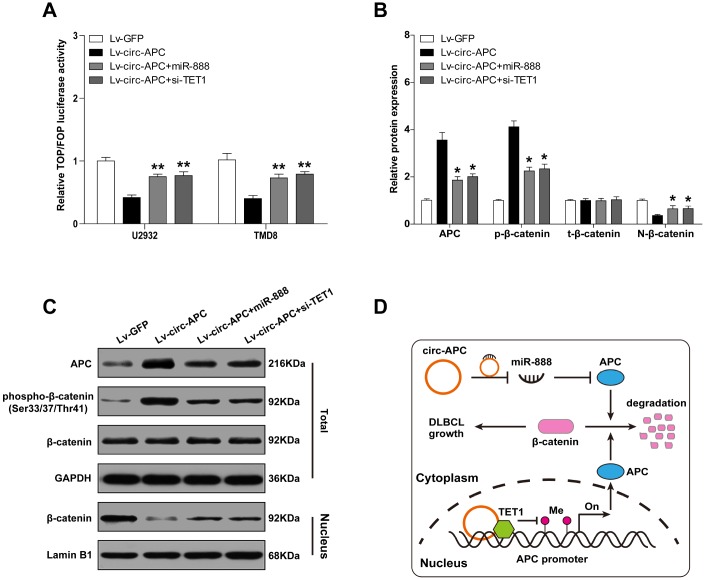
**Circ-APC dampens canonical Wnt/β-catenin signaling in DLBCL.** (**A**) TOPFlash/FOPFlash reporter assay in stably circ-APC-overexpressing U2932 and TMD8 cells transfected with miR-888 mimics or si-TET1. (**B** and **C**) Western blot analysis of the indicated proteins in stably circ-APC-overexpressing U2932 and TMD8 cells transfected with miR-888 mimics or si-TET1. (**D**) Schematic diagram of circ-APC inhibiting proliferation through the miR-888/*APC* and TET1/*APC* axes in DLBCL cells. *p < 0.05, **p < 0.01.

APC is a key inhibitor of the canonical Wnt/β-catenin pathway, and can increase the phosphorylation and subsequent proteolytic degradation of β-catenin, thus reducing its nuclear accumulation [16]. Therefore, we reasoned that circ-APC might counteract canonical Wnt/β-catenin signaling by elevating *APC* in DLBCL. To test this hypothesis, we performed a TOPFlash/FOPFlash reporter assay with wild-type and mutant TCF-4 consensus binding sites. As shown in Figure 6A, exogenous circ-APC expression dramatically reduced TOP/FOP transcriptional activity, but miR-888 overexpression or *APC* knockdown significantly prevented this repression. Circ-APC overexpression also markedly increased the expression of APC and the phosphorylation of β-catenin, but reduced the expression of nuclear β-catenin and well-known downstream targets of the Wnt/β-catenin pathway (c-MYC, cyclin D1, c-JUN, FGF4 and PPARD) (Figure 6B, 6C, Supplementary Figure 3). Exogenous expression of miR-888 or silencing of *APC* abrogated the above effects (Figure 6B, 6C). These results indicated that cytoplasmic and nuclear circ-APC can respectively bind to miR-888 and TET1 to co-upregulate *APC* expression, ultimately inactivating the oncogenic Wnt/β-catenin signaling pathway in DLBCL (Figure 6D).

### Circ-APC is an effective diagnostic and prognostic biomarker for DLBCL

**Figure 7:**
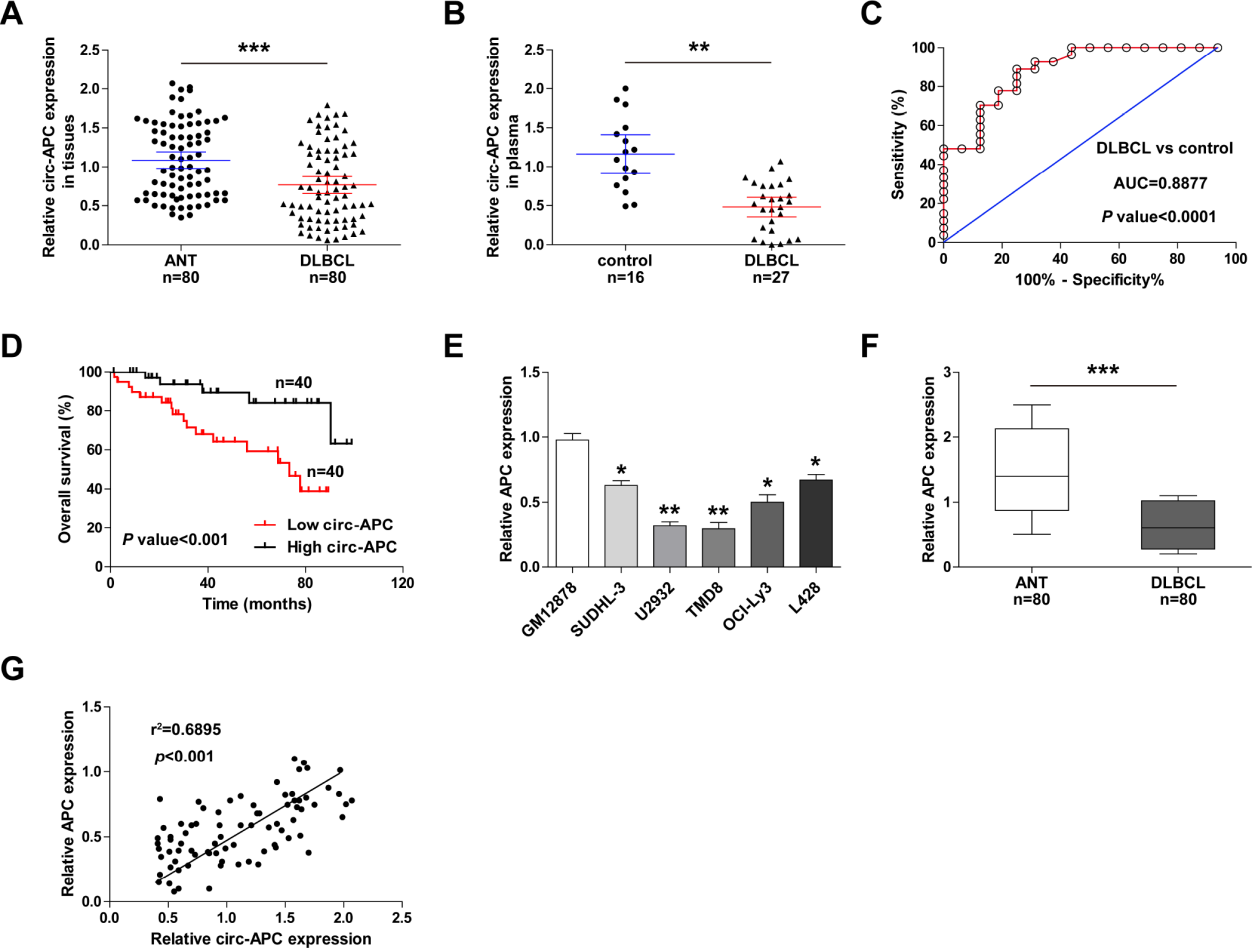
**Circ-APC is a promising diagnostic and prognostic biomarker in DLBCL.** (**A**) qRT-PCR analysis of circ-APC expression in 80 pairs of DLBCL and adjacent normal tissues. (**B**) qRT-PCR analysis of circ-APC expression in plasma from 16 healthy controls and 27 DLBCL patients. (**C**) Receiver operating characteristic curve analysis on the diagnostic utility of plasma circ-APC in DLBCL. (**D**) Kaplan-Meier plot displaying patients’ overall survival based on the median circ-APC level in 80 DLBCL tissues. (**E**, **F**) qRT-PCR analysis of *APC* expression in DLBCL cell lines and tissues. (**G**) The correlation between circ-APC and *APC* expression in DLBCL tissues. **p < 0.01, ***p < 0.001.

To determine the clinical applicability of circ-APC, we collected an additional 55 pairs of DLBCL and adjacent normal tissues for qRT-PCR analysis (a total of 80 pairs). Circ-APC expression was lower in DLBCL tissues than in normal tissues (Figure 7A). We also performed qRT-PCR to analyze circ-APC levels in plasma samples from 27 DLBCL patients and 16 healthy controls, and found that plasma circ-APC levels were significantly lower in DLBCL patients (Figure 7B). A receiver operating characteristic curve was used to assess the diagnostic utility of plasma circ-APC levels in DLBCL. As shown in Figure 7C, the area under the curve was 0.8877 (95% confidence interval = 0.8012-0.9651; *p* < 0.0001), suggesting that plasma circ-APC can excellently distinguish DLBCL patients from healthy controls.

We then evaluated the correlation of circ-APC expression with various clinicopathologic features. As shown in Table 1, DLBCL patients with lower circ-APC levels were more likely to exhibit an advanced Ann Arbor stage (*p* = 0.012), to resist CHOP-like (*p* = 0.004) and rituximab (*p* = 0.002) treatment, and to display a low International Prognostic Index (*p* = 0.032). A Kaplan-Meier plot revealed that the overall survival time was shorter in patients with lower circ-APC levels in their DLBCL tissues (Figure 7D). Cox multivariate analysis indicated that DLBCL tissue circ-APC expression was an independent prognostic factor for DLBCL patients (Table 2). In addition, *APC* mRNA expression was significantly downregulated in DLBCL cell lines and tissues (Figure 7E, 7F), and correlated strongly and positively with circ-APC expression (Figure 7G). These clinical data suggested that circ-APC expression is a useful indicator of DLBCL diagnosis and prognosis.

**Table 1:** Association of circ-APC expression with clinical parameters in DLBCL patients (n=80).

**Parameters**	**Total (n=80)**	**circ-APC expression**		***P* Value**
		**Low (n=40)**	**High (n=40)**	
Age				0.653
≤ 60	36	17	19	
> 60	44	23	21	
Gender				0.260
Male	45	20	25	
Female	35	20	15	
Ann Arbor stage				**0.012**
I-II	31	10	21	
III-IV	49	30	19	
Performance status				0.496
0-2	33	18	15	
3-4	47	22	25	
CHOP-like treatment				**0.004**
Response	39	13	26	
Non-response	41	27	14	
Rituximab				**0.002**
Response	34	10	24	
Non-response	46	30	16	
Subtype				0.822
GC	35	17	18	
Non-GC	45	23	22	
IPI				**0.032**
0-2	18	13	5	
3-5	62	27	35	

CHOP-like refers to CHOP (cyclophosphamide, adriamycin, vincristine and prednisone) or a CHOP-like regimen; GC: germinal centre-derived lymphoma; IPI: international prognostic index.

**Table 2:** Univariate and multivariate Cox proportional hazards regression model analysis of factors for OS in patients with DLBCL (n=80).

**Variable**	**Univariate analysis**		**Multivariate analysis**	
	**HR (95%CI)**	***P* value**	**HR (95%CI)**	***P* value**
Gender	1.023 (0.606−1.954)	0.634		
Age	1.253 (0.763−2.345)	0.227		
Ann Arbor stage	3.126 (1.635−5.247)	**0.006**	2.867 (1.578−4.729)	**0.015**
Performance status	1.542 (0.457−3.275)	0.075		
Subtype	1.745 (1.025−3.697)	0.086		
IPI	2.213 (1.468−4.358)	**0.034**	0.741 (0.586−2.357)	0.235
circ-APC expression	0.432 (0.236−0.701)	**0.001**	0.511 (0.374−0.837)	**0.008**

## DISCUSSION

As a special type of non-coding RNA with a loop structure, circRNA has opened a new chapter in cancer research. Numerous studies have linked circRNA to cancer initiation, development and progression [17]; however, to the best of our knowledge, this is the first study to explore the characteristics of circRNA in DLBCL. By evaluating paired DLBCL and normal tissues on a circRNA expression microarray, we identified substantially dysregulated circRNAs in DLBCL, suggesting that circRNA may also participate in DLBCL tumorigenesis.

We then focused on circ-APC, a previously undescribed circRNA derived from the back-splicing of *APC* exon 7 to exon 14. Circ-APC was significantly downregulated in DLBCL tissues, and this downregulation was associated with aggressive clinical features and a dismal prognosis. Exogenous circ-APC treatment suppressed DLBCL proliferation *in vitro* and *in vivo*. Through different mechanisms, cytoplasmic and nuclear circ-APC concurrently elevated the expression of the host gene *APC*, thereby inactivating canonical Wnt/β-catenin signaling and restraining DLBCL growth. These data shed light on the importance of circRNA in the pathogenesis of DLBCL.

Cellular sub-localization is essential for the biological functions of non-coding RNAs. Like long non-coding RNA, circRNA can extensively sponge miRNA in the cytoplasm. However, due to the highly stability of circRNA, its effects on miRNA may last longer than those of long non-coding RNA. We detected circ-APC in both the cytoplasm and the nucleus. Cytoplasmic circ-APC efficiently sponged miR-888, thereby reducing its repressive effects on *APC*. However, considering the partial nuclear localization of circ-APC, we inferred that circ-APC might also upregulate *APC* expression in the nucleus. Indeed, several studies have demonstrated that nuclear circRNAs govern the expression of their host genes by binding to proteins. As expected, we found that circ-APC could bind directly to TET1 (a well-known demethylase that iteratively oxidizes 5mC to 5hmC) and recruit it to the *APC* promoter, thereby reducing the methylation level and potentiating the transcription of *APC*. Thus, circ-APC upregulated *APC* expression in DLBCL at both the transcriptional and post-transcriptional levels. Through these effects on APC (a well-documented tumor suppressor that increases the phosphorylation and proteolytic degradation of β-catenin [16]), exogenous circ-APC suppressed canonical Wnt/β-catenin signaling.

The expression and function of circRNA appears to depend on the tissue and the disease stage [18]. For instance, circ-HIPK3 was reported as an oncogene in prostate cancer [19], hepatocellular carcinoma [20] and colorectal cancer [21], but as a tumor inhibitor in osteosarcoma [22], ovarian cancer [23] and bladder cancer [24]. Whether circ-APC is also an anti-cancer gene in other types of malignant tumors is worthy of further study.

Due to its high stability, circRNA is an excellent biomarker for human diseases, especially cancer [25]; examples include circ-SMARCA5 as a biomarker for hepatocellular carcinoma [26], circ-ANKS1B for breast cancer [27], circ-UBXN7 for bladder cancer [28] and circ-LMTK2 for gastric cancer [29]. Here, we confirmed the high stability of circ-APC, and further identified it as an independent protective factor in DLBCL. Circ-APC was even detectable in the plasma of DLBCL patients, where it was expressed at much lower levels than in healthy controls, suggesting its great potential as a non-invasive diagnostic biomarker for DLBCL. Large-scale studies of DLBCL samples are needed to authenticate this finding.

In summary, we have provided robust evidence that circ-APC suppresses the proliferation of DLBCL by inhibiting canonical Wnt/β-catenin signaling. Thus, the reactivation of circ-APC may be a feasible treatment option for this pernicious disease.

## MATERIALS AND METHODS

### CircRNA microarray expression profile

Differentially expressed circRNAs in DLBCL were identified through circRNA microarray analysis of three matched DLBCL and adjacent normal tissues on an ArrayStar Human circRNA chip (Rockville, MD, USA) at KangChen Biotech, Inc. (Shanghai, China). Total RNA was extracted and treated with RNase R to remove linear RNA. The remaining RNA was then reverse-transcribed to fluorescently labeled single-strand cDNA and hybridized to the array by standard protocols. The original data were normalized by RStudio software. The statistical significance of differential circRNA expression was established based on a fold-change >2 and a *p* value <0.05.

### DLBCL tissues, plasma and cell lines

In total, 80 pairs of DLBCL and para-cancerous tissues were collected from the Affiliated Cancer Hospital of Zhengzhou University. The detailed clinical parameters of the patients are described in Table 1. All surgically resected tissues were immediately placed in liquid nitrogen to avoid RNA degradation. Three of these pairs of tissues were used for the circRNA microarray analysis. In addition, plasma samples were obtained from 27 DLBCL patients and 16 healthy controls to evaluate the diagnostic utility of circ-APC for DLBCL. Each patient provided written informed consent prior to this study, and the study was approved by the Ethics Committee of the Affiliated Cancer Hospital of Zhengzhou University.

Human DLBCL cell lines (SUDHL-3, U2932, TMD8, OCI-Ly3 and L428) and normal human B lymphocytes (GM12878) were purchased from the Chinese Academy of Sciences (Shanghai, China). Mycoplasma detection was routinely performed before cell use.

### Polymerase chain reaction (PCR)

Total DNA and RNA were obtained with a DNA extraction kit (TIANGEN, Beijing, China) and TRIzol reagent (Invitrogen, CLD, CA, USA), respectively, and were quantified on a NanoDrop 2000 (Invitrogen). Cytoplasmic and nuclear RNAs were isolated with a PARIS™ Kit (Thermo Scientific) in accordance with the manufacturer's protocol. RNA was reverse-transcribed to cDNA with an MMLV-RT kit (Takara Bio, Otsu, Japan) with random hexamers based on the manufacturer’s instructions. Then, PCR was conducted, and the products were subjected to nucleic acid electrophoresis on a 2% agarose gel.

For qRT-PCR, SYBR Premix EX Taq (Takara Bio) was used in a final reaction volume of 10 μL. Relative gene expression was calculated by the 2^−ΔΔCT^ method. The sequences of the primers used in this study are shown in Table 3.

**Table 3:** The primer sequences for the analyzed genes.

**Gene**	**Direction**	**Sequence (5′- 3′)**
Circ-APC (divergent)	Forward	TGCGAGAAGTTGGAAGTGTG
	Reverse	TCTCTGCTTCTGTTGCTTGG
Circ-APC (convergent)	Forward	GGCAGAATGAAGGTCAAGGA
	Reverse	CACCATTTCCACCTTGGTTC
APC	Forward	AAGAAGCTCTGCTGCCCATA
	Reverse	GGATTCAATCGAGGGTTTCA
GAPDH	Forward	ACCCAGAAGACTGTGGATGG
	Reverse	TTCAGCTCAGGGATGACCTT
miR-1183	Forward	ACTGACCACTGTAGGTGATGGT
	Reverse	GCGAGCACAGAATTAATACGACTCACTATAGG
miR-490-5p	Forward	CATGGATCTCCAGGTGG
	Reverse	TGGTGTCGTGGAGTCG
miR-1206	Forward	CAGTGTTCATGTAGATGTTTAAGCTCTTG
	Reverse	GCATAATTTGGCAGCGTTCA
miR-330-3p	Forward	GCAGAGATTCCGTTGTCGT
	Reverse	GCGAGCACAGAATTAATACGAC
miR-421	Forward	CTCACTCACATCAACAGACATTAATT
	Reverse	GTGCAGGGTCCGAGGT
miR-1252	Forward	GCGCAGAGAAGGAAATTGA
	Reverse	GGTCCAGTTTTTTTTTTTTTTTAAATGA
miR-888	Forward	ATGTGGCAGATCCCACAGGAGTTT
	Reverse	ACTGGGTTTGACTTCGTAGCCCTT
miR-7	Forward	TGGAAGACTAGTGATTTTGTTGT
	Reverse	CAGTGCGTGTCGTGGAGT
miR-661	Forward	ACACTCCAGCTGGGTGCCTGGGTCTCTGGCCT
	Reverse	CTCAACTGGTGTCGTGGA
miR-924	Forward	TGCGGAGAGTCTTGTGATGTC
	Reverse	CAGTGCGTGTCGTGGAGT
miR-1257	Forward	GGATCTTCGAGAAATCCGG
	Reverse	AGGCATTGGCATTGACTAAC
miR-1298	Forward	ACACTCCAGCTGGGTTCATTCGGCTGTCCA
	Reverse	TGGTGTCGTGGAGTCG
U6	Forward	CGCTTCACGAATTTGCGTGTCAT
	Reverse	GCTTCGGCAGCACATATACTAAAAT

### Cell transfection and generation of stably circ-APC-overexpressing cell lines

Cells were transfected with Lipofectamine 3000 (Invitrogen) according to the manufacturer’s instructions. Control miRNA or miR-888 mimics (5′-UACUCAAAA AGCUGUCAGUCA-3′), along with negative control small interfering RNA (si-NC), si-TET1 (5′-GCACGCAT GAATTTGGATA-3′), or si-APC (5′-CGGGAAGGAUC UGUAUCAA-3′) were obtained from GenePharma Co., Ltd (Shanghai, China). The overexpression plasmids for *TET1*, *TET2* and *TET3* were constructed through the insertion of their full-length sequences into pcDNA 3.0 vectors (Invitrogen). The cell transfection efficiency was assessed by qRT-PCR after 48 hours of transfection.

For stable circ-APC overexpression, the full-length sequence of circ-APC was embedded in the PLCDH-ciR lentiviral vector (Geneseed, Guangzhou, China). Then, U2932 and TMD8 cells were infected with these lentiviral particles, and stable cells were selected with 1.5 μg/mL puromycin for 72 hours. The infection efficiency was determined by fluorescence microscopy and qRT-PCR analysis.

### Cell proliferation assay

The proliferative abilities of U2932 and TMD8 cells were assessed by CCK-8, EdU DNA incorporation and cell cycle assays. For the CCK-8 assay, cells were cultured for the indicated times and then treated with 10 μL of the CCK-8 solution (Dojindo, Kumamoto, Japan). The absorbance was measured at 450 nm after two hours of CCK-8 treatment. The DNA incorporation rate was tested with a Cell-Light EdU Apollo567 *In Vitro* Kit (RiboBio, Guangzhou, China) in accordance with the manufacturer’s protocols. For the evaluation of the cell cycle, U2932 and TMD8 cells were fixed and stained with a 20-mg/mL propidium iodide (Beyotime, Shanghai, China) solution, and a flow cytometer was used to detect the proportion of cells in the G0/G1, S and G2/M phases.

### Animal study

To determine the function of circ-APC *in vivo*, we established a xenograft tumor model by injecting control or stably circ-APC-overexpressing U2932 cells into the subaxillary region of nude mice (n=6 per group). The volume of each subcutaneous tumor was measured every week. Thirty-five days after the injection, the mice were sacrificed, and their tumors were dissected and weighed. Then, the tumor tissues were analyzed by qRT-PCR for circ-APC or *APC* mRNA expression, and by Western blotting for APC protein expression. The animal study was conducted in accordance with the procedures of the Animal Care Committee of the Affiliated Cancer Hospital of Zhengzhou University.

### Western blot assay

Total protein was isolated from cells and nude mouse tissues with radioimmunoprecipitation assay lysis buffer (Beyotime). The proteins were separated by 10% sodium dodecyl sulfate polyacrylamide gel electrophoresis, transferred onto a polyvinylidene difluoride membrane and blocked with a 5% nonfat dry milk solution. The membrane was then incubated with one of the following primary antibodies: anti-APC (1:1000, #ab40778, Abcam, UK), anti-phosphorylated-β-catenin (1:500, #9561, Cell Signaling Technology, USA), anti-TET1 (1:3000, #GT1462, Invitrogen, USA) and anti-β-catenin (1:1000, #8480, Cell Signaling Technology, USA). Anti-GAPDH (1:10000, #60004-1-Ig, Proteintech, USA) and Lamin B1 (1:2500, #13435, Cell Signaling Technology, USA) antibodies were used as internal references for total and nuclear proteins, respectively. Lastly, the membrane was incubated with a horseradish peroxidase-conjugated rabbit or mouse secondary antibody, and was developed with an enhanced chemiluminescence solution (Beyotime).

### RNA pull-down assay

For the detection of miRNAs pulled down by circ-APC, U2932 and TMD8 cell lysates were collected and incubated overnight with a biotinylated control (5′-GCTAAAGTCAAGTCTGAAAAGCAATGATGTTGTCCACTGG-3′) or circ-APC probe (5′-AAAAGCGAAG AATAGCCAGAA-3′) from RiboBio. Next, the lysates were incubated with streptavidin magnetic beads (Invitrogen) at room temperature for three hours to generate bead/circ-APC complexes. The RNA bound by circ-APC was extracted with TRIzol solution, purified with an RNeasy Mini Kit (Qiagen, Dusseldorf, Germany) and analyzed by qRT-PCR to determine the expression of the 12 indicated miRNAs. In addition, the proteins bound by circ-APC were collected in radioimmunoprecipitation assay lysis buffer, and Western blot analysis was performed to assess TET1 expression.

For the detection of miR-888 pulling down circ-APC, biotinylated wild-type or mutant miR-888 mimics were synthesized and transfected into U2932 and TMD8 cells with Lipofectamine 3000 (Invitrogen). The cells were then incubated with the above streptavidin magnetic beads, and circ-APC expression was detected by qRT-PCR.

### Luciferase reporter and TOPFlash/FOPFlash reporter assays

The ability of miR-888 to bind to circ-APC or *APC* was assessed with a luciferase reporter assay. First, pmirGLO vectors (Promega, WI, USA) containing full-length circ-APC or *APC* sequences with wild-type or mutant miR-888 binding sites were synthesized. Then, Lipofectamine 3000 (Invitrogen) was used to co-transfect U2932 and TMD8 cells with the above wild-type or mutant reporters and control miRNA or miR-888 mimics. After 48 hours of transfection, the cells were harvested, and the luciferase activity was detected with a Dual-Luciferase Reporter Assay System (Promega) in accordance with standard protocols.

For the detection of transcriptional activity in the Wnt/β-catenin signaling pathway, circ-APC-overexpressing U2932 and TMD8 cells were co-transfected with a TOPFlash/FOPFlash reporter (Addgene, MA, USA) and miR-888 mimics or si-TET1. Then, the luciferase activity was assessed based on the ratio of TOPFlash to FOPFlash.

### RNA immunoprecipitation (RIP)

The potential binding between TET1 and circ-APC was assessed by an RNA immunoprecipitation assay with a Magna RIP™ kit (Millipore, Schwalbach, Germany) and an anti-TET1 antibody (1:500, #GT1462, Invitrogen, USA). In brief, U2932 and TMD8 cell lysates were harvested, incubated at 4°C overnight with an anti-TET1 antibody and subsequently incubated with protein A/G magnetic beads at 25°C for two hours. Then, TET1-bound RNA was isolated from the above complexes with TRIzol solution, purified with an RNeasy Mini Kit (Qiagen) and analyzed by qRT-PCR for circ-APC expression.

### Chromatin isolation by RNA purification (ChIRP) and chromatin immunoprecipitation (ChIP) assays

Chromatin was prepared by standard protocols. Briefly, U2932 and TMD8 cells were harvested, cross-linked with 1% formaldehyde for 10 minutes and quenched with glycine for five minutes at room temperature. Then, at room temperature, micrococcal nuclease was used to digest the genome into fragments with an average length of 200-1000 bp.

For the ChIRP assay, the chromatin fragments thus prepared were incubated with a biotinylated control or circ-APC probe overnight at 4°C. The fragments were then incubated with streptavidin magnetic beads, and the enrichment of circ-APC with the *APC* promoter region was assessed by qRT-PCR.

For the ChIP assay, the prepared chromatin fragments were incubated with an anti-TET1 (1:500, #GT1462, Invitrogen, USA) or anti-5hmC (1:200, #ab106918, Abcam, UK) antibody overnight at 4°C, and were subsequently incubated with protein A/G magnetic beads (Invitrogen). Then, the precipitated DNA was eluted and purified with a DNeasy Mini Kit (Qiagen), and the enrichment of TET1 or 5hmC with the *APC* promoter region was assessed by qRT-PCR.

### Data analysis

All data are presented as the mean ± standard deviation (SD) of three replicates. Student’s t-tests or Chi-square tests were used as appropriate to determine the differences between groups. Receiver operating characteristic curve and Kaplan-Meier analyses were respectively employed to identify the diagnostic and prognostic value of circ-APC in DLBCL. All the statistical analyses in this study were two-tailed and were conducted with SPSS 22.0 software. *P* values ≤ 0.05 were considered significant. *p < 0.05, **p < 0.01, ***p < 0.001.

## Supplementary Material

Supplementary Figures
