# Supplementary material for: A circular RNA from APC inhibits the proliferation of diffuse large B-cell lymphoma by inactivating Wnt/β-catenin signaling via interacting with TET1 and miR-888
Source: Aging (Albany NY). 2019 Oct 13;11(19):8068–84. doi: 10.18632/aging.102122 (PMC6814595; doi:10.18632/aging.102122)
Supplement: Supplementary Figures [file aging-11-102122-s001.pdf]

SUPPLEMENTARY FIGURES

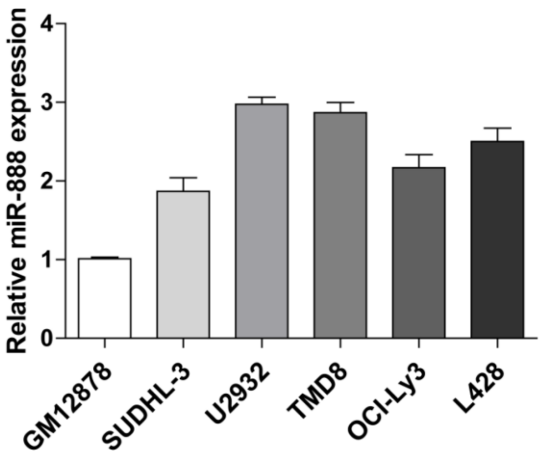

Supplementary Figure 1. qRT-PCR analysis for miR-888 expression in five DLBCL cell lines and one human normal B lymphocyte GM12878 cells.

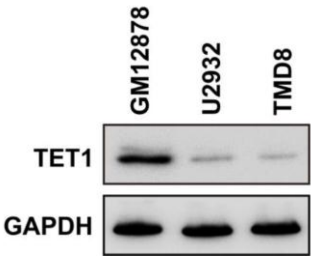

Supplementary Figure 2. Western blotting analysis for TET1 protein expression in the indicated cell lines. GAPDH was used as loading control reference.

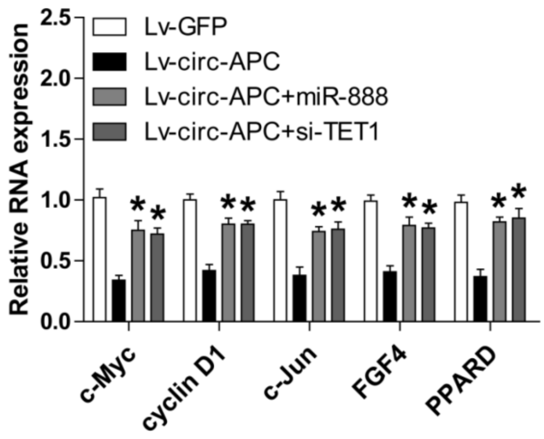

Supplementary Figure 3. qRT-PCR analysis for the expression of the downstream target genes of Wnt/β-catenin pathway. *$p < 0.05$.
